# Protective Behaviour of Citizens to Transport Accidents Involving Hazardous Materials: A Discrete Choice Experiment Applied to Populated Areas nearby Waterways

**DOI:** 10.1371/journal.pone.0142507

**Published:** 2015-11-16

**Authors:** Esther W. de Bekker-Grob, Arnold D. Bergstra, Michiel C. J. Bliemer, Inge J. M. Trijssenaar-Buhre, Alex Burdorf

**Affiliations:** 1 Department of Public Health, Erasmus MC, University Medical Centre Rotterdam, Rotterdam, The Netherlands; 2 The Zeeland Public Health Service, Goes, The Netherlands; 3 Institute of Transport and Logistic Studies, University of Sydney Business School, Sydney, New South Wales, Australia; 4 Netherlands Organisation for Applied Scientific Research, Utrecht, The Netherlands; Duke University Medical Center, UNITED STATES

## Abstract

**Background:**

To improve the information for and preparation of citizens at risk to hazardous material transport accidents, a first important step is to determine how different characteristics of hazardous material transport accidents will influence citizens’ protective behaviour. However, quantitative studies investigating citizens’ protective behaviour in case of hazardous material transport accidents are scarce.

**Methods:**

A discrete choice experiment was conducted among subjects (19–64 years) living in the direct vicinity of a large waterway. Scenarios were described by three transport accident characteristics: odour perception, smoke/vapour perception, and the proportion of people in the environment that were leaving at their own discretion. Subjects were asked to consider each scenario as realistic and to choose the alternative that was most appealing to them: staying, seeking shelter, or escaping. A panel error component model was used to quantify how different transport accident characteristics influenced subjects’ protective behaviour.

**Results:**

The response was 44% (881/1,994). The predicted probability that a subject would stay ranged from 1% in case of a severe looking accident till 62% in case of a mild looking accident. All three transport accident characteristics proved to influence protective behaviour. Particularly a perception of strong ammonia or mercaptan odours and visible smoke/vapour close to citizens had the strongest positive influence on escaping. In general, ‘escaping’ was more preferred than ‘seeking shelter’, although stated preference heterogeneity among subjects for these protective behaviour options was substantial. Males were less willing to seek shelter than females, whereas elderly people were more willing to escape than younger people.

**Conclusion:**

Various characteristics of transport accident involving hazardous materials influence subjects’ protective behaviour. The preference heterogeneity shows that information needs to be targeted differently depending on gender and age to prepare citizens properly.

## Introduction

The transport of hazardous material is an economic activity of concern to society [1]. A hazardous material can be defined as a substance or material determined to be capable of posing an unreasonable effect to health, safety or property when transported [2]. Hazardous materials are transported by road, rail, water, air and pipeline. Although the probability of a hazardous material transport accident is small, the consequences may be severe for humans and environment [1].

The literature shows that an individual with a high perceived risk of harm would be motivated to take action to reduce his or her health risk [3,4]. Possible and useful actions, that can be taken by the general public to reduce health consequences of a hazardous material transport accident, are for instance seeking shelter (i.e., go inside a building) or escaping the transport accident area (i.e., leaving at their own discretion). However, members of the general public often misinterpret their risk of health problems [5]. Based on the behaviour motivation theory, which describes the effects of perceptions of risk on behaviour, correcting these misinterpretations is seen as a way to encourage appropriate behaviour [5]. To improve the information for and preparation of citizens at risk to hazardous material transport accidents, a first important step is to investigate how different characteristics of hazardous material transport accidents will influence citizens’ protective behaviour.

A broad range of studies exists that investigates citizens’ protective behaviour in case of disasters/accidents [6–20]. However, most of these studies are qualitative studies or studies that contain limited choices or scenarios of disasters/accidents, which limits the comparability between these studies to develop guidance for protective action of citizens.

Therefore the aim of this study is to quantify how different characteristics of hazardous material transport accidents will influence protective behaviour of the general public. We hereto use a stated preference technique by conducting a discrete choice experiment (DCE) focusing on hypothetical hazardous material transport accidents at a river in a populated area in the Netherlands. The motivation to conduct this DCE study is two-fold: 1) There is a great lack of quantitative research with respect to hazardous material transport; and 2) Conducting a DCE (a quantitative method) will enhance the knowledge gap where qualitative studies fall short; that is, based on this DCE study the protective behavior of citizens for a whole range of transport accidents involving hazardous materials can be determined (at least for populated areas nearby waterways), which is useful to develop guidance for protective action of citizens.

## Materials and Methods

### 2.1. Discrete Choice Experiment

**Fig 1:**
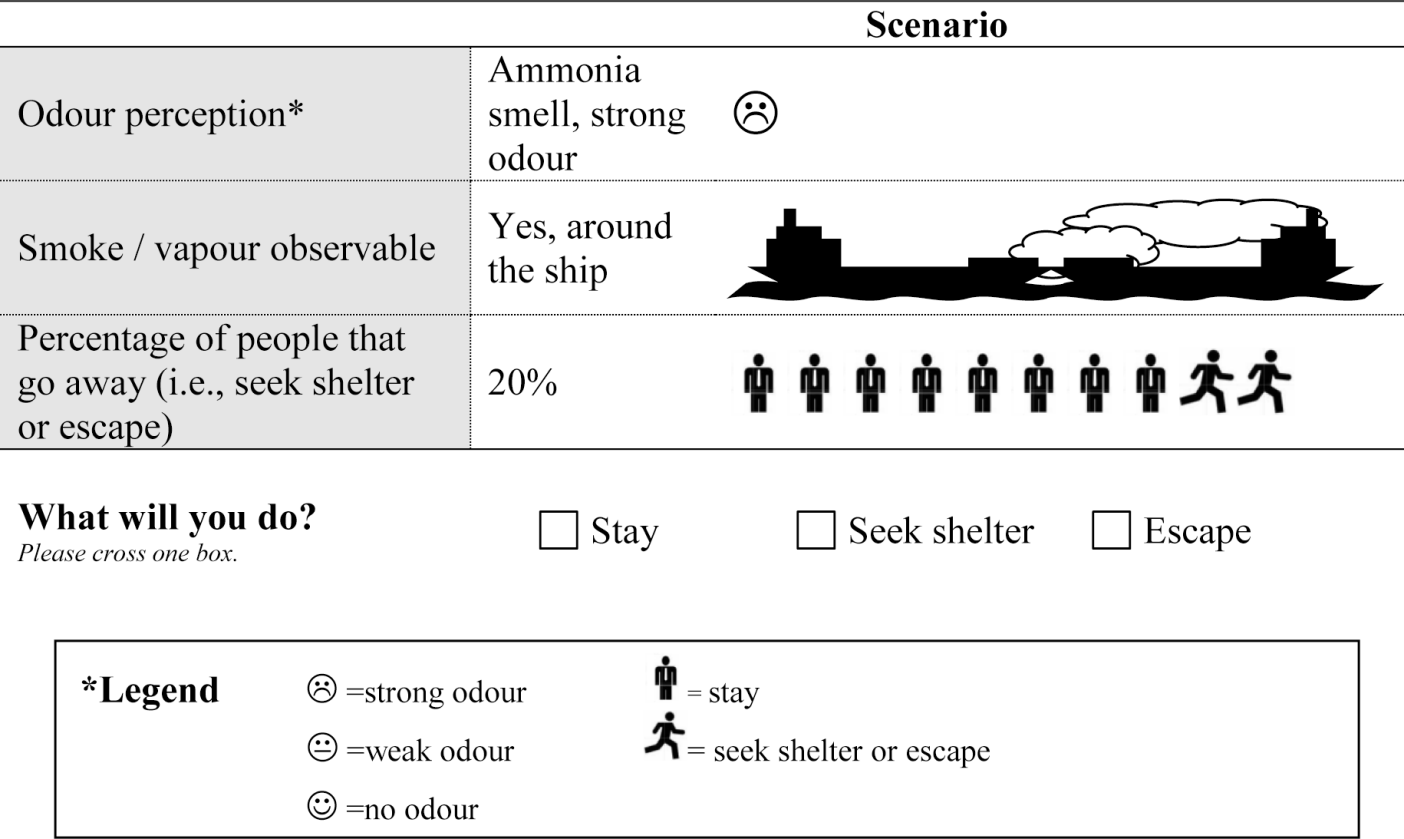
Example of a choice set.

Discrete choice experiment (DCE) is a quantitative technique to investigate individual preferences (choice behaviour). The DCE has a solid foundation in random utility theory [21,22] and includes a Nobel prize-winning econometric approach [23]. Within a DCE, subjects are presented with several choice sets (see Fig 1 for a choice set example). In each choice set subjects are asked to choose between two or more alternatives. The alternatives are described by its characteristics (attributes) [24]. Those attributes are further specified by variants of that attribute (attribute levels). It is assumed that the subject’s preference for an alternative is determined by the levels of those attributes [24]. Resulting choices reveal an underlying utility function. The stated preferences allow the utility calculation of *all* alternatives (i.e. all permutations of attribute levels, including alternatives that are not presented to respondents). The DCE technique is mainstream in marketing, transport and environmental economics, where it is used to predict individual and collective choices. Its application in healthcare and public health has grown exponentially as the method is easy to apply and appears efficient at first glance [25,26]. The DCE approach combines (i) consumer theory [27] and (ii) random utility theory [28], which both assume that an individual acts rationally and always chooses the alternative with the highest satisfaction (i.e., utility), as well as (iii) experimental design theory and (iv) econometric analysis [26]. See Louviere *et al*. [29]; Hensher *et al*. [30]; Rose and Bliemer [31]; Lancsar and Louviere [32]; and Ryan *et al*. [33] for further details on conducting a DCE and its theoretical issues.

### 2.2. Attributes and attribute levels

We followed recent guidelines for DCE practice [32,34]. A focus group study and literature [1,35] were used to obtain insights into relevant attributes and attribute levels to be included in the DCE regarding protective behaviour of citizens to transport accidents involving hazardous materials. For the focus group study, we conducted two focus group discussions with the general population living in the direct vicinity of a large waterway in the Netherlands (six and seven participants, respectively). A topic list based on the Protective Action Decision Model [35] was created to structure the discussions on protective behaviour of citizens regarding transport accidents involving hazardous materials. As a result, three transport accident attributes were selected: odour perception, smoke/vapour perception, and the proportion of people in the environment that are leaving at their own discretion. We aimed at selecting a sufficient wide range of attribute levels that could be realistic in the near future and were plausible and understandable for respondents (Table 1). In this research ammonia (toxic) and propane gas (explosive/flammable) were selected as hazardous materials because ammonia and propane (odorized by mercaptan) are transported across the river in large quantities.

**Table 1:** Considered attributes and attribute levels.

Attributes	Levels
Odour perception	None (reference level)
	Ammonia, weak odour
	Ammonia, strong odour
	Mercaptan, weak odour
	Mercaptan, strong adour
Smoke/vapour perception	None (reference level)
	Yes, around the ship
	Yes, towards the beach/quay
Proportion of people that are leaving	0%
	20%
	50%
	80%

### 2.3. Study design and questionnaire

The combination of the attributes and levels (Table 1) resulted in 60 possible/hypothetical transport accident scenarios. As fatigue of filling out a lengthy questionnaire prohibited presenting all scenarios to a single individual (i.e., a full factorial design), a subset of scenarios was required (i.e., a fractional factorial design) [36]. For the final selection of the scenarios to be presented in the questionnaire (i.e., the scenarios in which the respondent was asked to choose between three discrete protective behaviour options: staying, seeking shelter, or escaping (see Fig 1, for an example of a so-called choice set)), we generated a D-efficient DCE design [36, 37] using Ngene software (version 1.1.1, http://www.choice-metrics.com/). In order to generate such an efficient design, prior values for the behavioural coefficients of each of the attributes in the utility function were needed. To this end we conducted a pilot study (N = 40) first to collect prior information and to obtain insights in the feasibility and validity of the questionnaire and DCE data. Based on the pilot study (data not shown) we concluded that the target group (general population) was able to understand the DCE, could manage the length of the questionnaire, did not become confused by any unlikely combination of attribute levels, and had no difficulties in completing 12 choice sets of the DCE task.

The choice results from the pilot study (data not shown) were used to estimate behavioural coefficients to each of the attributes, which were used to locate an even more efficient DCE design for the main study (i.e., a design that had smaller standard errors [31,38], thereby increasing the reliability of the estimation of the behavioural coefficients). With this design, resulting in 24 scenarios, we were able to estimate all scenario specific main effects (i.e., to determine what influence the transport accident attributes had on citizens’ protective behaviour (staying, seeking shelter, or escaping)). Presenting a single individual with 24 choice sets was expected to result in a low return rate for the questionnaire and partially completed questionnaires [39]. Therefore, in order to reduce the burden on respondents and to take the pilot results into account, the 24 choice sets were blocked [30] into two sets of 12 choice tasks each. Each questionnaire started with detailed information about the visible accident (i.e. setting the scene) and the protective behaviour alternatives (S1 File). The main part of each questionnaire—randomly assigned to respondents—comprised a block of 12 choice sets. In each choice set, subjects were asked to consider the specific transport accident scenario as realistic and to choose the alternative that was most appealing to them: staying, seeking shelter, or escaping (see Fig 1 for an example of a choice set). The questionnaire further contained questions on background variables (e.g. age, gender, educational level, marital status) and a question assessing experienced difficulty of the questionnaire (5-point scale) (S1 File; the complete questionnaire is available from the authors on request).

### 2.4. Study sample and elicitation mode

A representative sample of 1,994 subjects aged 19–64 years was randomly recruited using the population registry of two cities (Vlissingen and Terneuzen) in the direct vicinity of a large waterway (Westerschelde) in the Southwest of the Netherlands. This population registry contains 31,907 subjects aged 19–64 years. Hence, our representative sample, agrees with a quota of 6.2%. We used age, gender and civil status as variables to guarantee representativeness. Using a rule of thumb as suggested by Orme [40], we strived to include at least 600 respondents. Subjects received an invitation letter and questionnaire by mail, and could return the questionnaire in a postage-paid envelope that was included in the mailing package. Due to practical reasons and positive past experiences, the survey was administered with the return of a mail survey as the only option presented for its completion. Two reminders were sent 1.5 and three weeks later in case of non-response. The information to participants explained that by filling out the questionnaire informed consent was given (S2 File). Under the Dutch law for the agreement on medical treatment, questionnaire surveys are not subject to approval by an institutional ethics committee. However, the Law for Protection of Personal Data requires informed consent and also procedures for the protection of personal privacy. These procedures are laid done in the Code of Conduct for Medical research (at www.federa.org), established by the Council of the Federation of Medical Scientific Societies. In this research project we have strictly adhered to these procedures and the data were analysed anonymously (in more detail, the data were anonymized prior to we–as authors—receiving them, and we–as authors—did not have access to participant names and addresses).

### 2.5. Statistical analyses

The DCE contained three dependent variables for each choice set. The DCE was analysed by taking each choice (i.e., protective behaviour options staying, seeking shelter, or escaping) made in every choice set as an observation. Taking our interest in preference heterogeneity into account as well as our sample size, a mixed logit model or a latent class model were both good alternatives to analyse the choice observations. Using the Nlogit software (http://www.limdep.com/) and taking the best model fit into account, the observations were analysed by a panel mixed logit model in error component form [41], also called error component model. The error component model is a parsimonious specification of the mixed logit model, where preference heterogeneity is modelled for only a specific set of variables. In our error component model, we capture preference heterogeneity for the possible behavior reactions. Hence, the alternative specific constants for types of reaction ‘hide’ and ‘escape’ were classified as random (assuming a normal distribution), while preferences for the other attributes were assumed to be homogeneous across respondents in the sample. The advantage of using an error component model over a mixed logit model with random coefficient for all attributes is that it can capture the most important elements of preference heterogeneity in a rather parsimonious way. The final specifications of the utility functions used were:

$$\begin{array}{ll} {\text{V}}_{\text{seeking shelter}} & =\beta_{0}+\beta_{1}\;\text{odour}\_\text{ammonia}\_\text{weak}+\beta_{2}\text{odour}\_\text{ammonia}\_\text{strong}+\\ \; & \beta_{3}\text{odour}\_\text{mercaptan}\_\text{weak}+\beta_{4}\text{odour}\_\text{mercaptan}\_\text{strong}+\\ \; & \beta_{5}\text{smoke}\_\text{vapour}\_\text{ship}+\beta_{6}\text{smoke}\_\text{vapour}\_\text{beach}+\beta_{7}\text{leaving}\_\text{people}\\ {\text{V}}_{\text{escaping}} & =\beta_{8}+\beta_{9}\text{odour}\_\text{ammonia}\_\text{weak}+\beta_{10}\text{odour}\_\text{ammonia}\_\text{strong}+\\ \; & \beta_{11}\text{odour}\_\text{mercaptan}\_\text{weak}+\beta_{12}\text{odour}\_\text{mercaptan}\_\text{strong}+\\ \; & \beta_{13}\text{smoke}\_\text{vapour}\_\text{ship}+\beta_{14}\text{smoke}\_\text{vapour}\_\text{beach}+\beta_{15}\text{leaving}\_\text{people}\\ {\text{V}}_{\text{staying}} & =0 \end{array}$$ (Eq. 1)

where

$$\begin{array}{ll} V & \;\text{represent the systematic utility}\;\left(\text{stated preference}\right)\;\text{that a respondent has}\\ \; & \;\text{for a protective behaviour option}; \end{array}$$

$$\begin{array}{ll} \beta_{0,}\beta_{8} & \;\text{represent alternative specific constants for a protective behaviour}\\ \; & \;\text{option}\;\left(‘\text{staying}’\;\text{acts as the reference level}\right); \end{array}$$

$$\begin{array}{ll} \beta_{1-7\;,}\beta_{9-15} & \;\text{represent alternative specific parameter weights}\;\left(\text{or coefficients}\right)\\ \; & \;\text{associated with the attribute}\left(\text{level}\right)\text{s}:\;\text{odour perception},\;\text{smoke}/\text{vapour}\\ \; & \;\text{perception},\;\text{and the proportion of people in the environment that are}\\ \; & \;\text{leaving at their own discretion}. \end{array}$$

The value of each coefficient represents the importance that respondents assign to a certain attribute or attribute level. However, different attributes utilise different units of measurement. For example, the coefficient ‘the proportion of people in the environment that are leaving at their own discretion’ represents the importance per 10% of people in the environment that are leaving at their own discretion. That is, in a scenario where 50% of people in the environment are leaving at their own discretion, the coefficient should be multiplied by five in order to establish its relative contribution to utility compared to other attributes.

In addition to the parameter estimates, the estimation procedure also allows for tests of statistical significance. Statistical significance of a coefficient (p-value ≤0.05) indicates that respondents considered the attribute (or attribute level) important in making their choices (i.e., protective behaviour) in the DCE. The sign of the coefficient reflects whether the attribute (level) has a positive or negative effect on the specific stated protective behaviour option (utility). A priori we expected all attributes to be important, and that the attribute ‘the proportion of people in the environment that are leaving at their own discretion’ and stronger ammonia/mercaptan odour levels would have a positive effect on leaving the transport accident area. Additionally, sensitivity analyses were conducted to test whether specific respondent characteristics (e.g., age, gender, educational level) had a significant influence on the protective behaviour option made.

We also calculated the choice probabilities (the mean protective behaviour) to provide a way to convey DCE results to policy makers that are more easily understandable. We calculated the choice probabilities for a mild looking transport accident (i.e., a visible transport accident involving hazardous materials on a populated waterway without any odour perception or smoke/vapour perception, and where 0% of the people in the environment will leave the location) as well as for a severe looking transport accident (i.e., a visible transport accident involving hazardous materials on a populated waterway with a strong ammonia odour perception, smoke/vapour perception towards the beach/quay, and where 80% of the people in the environment will leave the location). The mild and severe looking accidents–representing the bookends of the spectrum for hazardous accidents—were chosen to help the reader in understanding the usefulness of such an analysis, and to show how these different accidents lead to a different protective behavior (stay, hide, or escape) of citizens. The choice probabilities were simulated based on the panel error component coefficients using 1,000 pseudo-random draws from the probability density functions estimated for the random error components.

## Results

### 3.1. Respondents

The response to the questionnaire was 44% (881/1,994). Respondents had a mean age of 47 years (SD = 12), about half of the respondents were male, 26% had a high educational level, and they lived predominantly together with a partner (Table 2).

**Table 2:** Characteristics of respondents who completed the discrete choice experiment survey (N = 881).

	Sample statistics	
	Mean	SD
Age (years)	47	12
	N	%
Age group (years)		
18–29	96	11
30–39	128	15
40–49	221	25
50–59	275	31
60–64	151	17
missing	10	1
Gender		
Male	445	51
Female	431	49
missing	5	1
Educational level		
Low	304	35
Average	343	39
High	227	26
missing	7	1
Civil status		
Married, registered partnership	636	72
Unmarried	166	19
Divorced	62	7
Widow / widower	17	2

### 3.2. DCE results

Weak odours did not influence respondents’ protective behaviour to seek shelter more compared to ‘no odour perception’ (Table 3; p = 0.58 and p = 0.10 for weak ammonia and mercaptan, respectively). However, strong odours made respondents seek shelter more (p<0.001), with ammonia having a stronger effect (coefficient values of 1.19 and 0.59 for strong ammonia and strong mercaptan odour perception, respectively; Table 3). For escape, which was much more preferred than seeking shelter (coefficient values of 1.90 and -0.42 respectively), any odour seemed to be important and very much odour made respondents want to escape. Smoke did not seem relevant for respondents to seek shelter or escape more, unless it came close to them. The transport accident attribute ‘proportion of people that are leaving’ proved to influence respondent’s protective behaviour to seek shelter and escape more (Table 3). The positive sign of this coefficient showed that the more people in the environment were seeking shelter or leaving at their own discretion, the more willing the respondent was to leave the transport accident area as well. The estimated standard deviations for the protective behaviour options ‘seeking shelter’ and ‘escaping’ were significant, which indicated stated preference heterogeneity among subjects for these protective behaviour options.

**Table 3:** The influence of characteristics of hazardous material transport accidents on citizens’ protective behaviour based on a panel error component model (n = 881).

Alternative specific constant		Coefficient	s.e.	p-value
Type of reaction				
Stay (reference level)		0		
Seek shelter	mean	-0.423	0.115	<0.001
	s.d.	2.169	0.101	<0.001
Escape	mean	1.904	0.103	<0.001
	s.d.	2.346	0.089	<0.001
Attributes Seek shelter		Coeff	s.e.	p-value
Odour perception				
None (reference level)		-1.587		
Ammonia, weak odour		-0.048	0.087	0.582
Ammonia, strong odour		1.189	0.109	<0.001
Mercaptan, weak odour		-0.144	0.087	0.100
Mercaptan, strong odour		0.589	0.112	<0.001
Smoke/Vapour perception				
None (reference level)		-0.812		
Yes, around the ship		0.116	0.072	0.104
Yes, towards the beach/quay		0.696	0.074	<0.001
Proportion of people that are leaving (per 10%)		0.130	0.018	<0.001
Attributes Escape		Coeff	s.e.	p-value
Odour perception				
None (reference level)		-2.366		
Ammonia, weak odour		-0.345	0.076	<0.001
Ammonia, strong odour		1.656	0.101	<0.001
Mercaptan, weak odour		-0.325	0.076	<0.001
Mercaptan, strong odour		1.380	0.101	<0.001
Smoke/Vapour perception				
None (reference level)		-1.229		
Yes, around the ship		0.032	0.063	0.609
Yes, towards the beach/quay		1.197	0.067	<0.001
Proportion of people that are leaving (per 10%)		0.206	0.016	<0.001
Model fit				
Log likelihood		-6,064		
AIC		1.164		
Pseudo R-squared		0.472		

Notes: (1) effect coded variables used for odour perception and smoke/vapour perception; (2) normal distribution for random coefficient used on constants (i.e. ‘type of reaction’); (3) the value of the reference levels of the categorical attributes equals the negative sum of the coefficients of the included attributes; (4) s.e. = standard error; (5) S.D. = standard deviation; and (6) 10,451 observations (881 subjects x 12 choice sets would result in 10,572 observations. However, 121 oberservations were missed because some respondents did not fill in one or more choice sets); AIC = Akaike information criterion

The sensitivity analysis showed that two characteristics of subjects had a significant influence on the stated preference for seeking shelter and escaping (see S1 Table). That is, males were less willing to seek shelter than females, and elderly people were more willing to escape than younger people.

In case of a mild looking transport accident involving hazardous materials at a river in a populated area, based on our DCE results the predicted probability that a subject would stay, seek shelter or escape was 62%, 13%, and 25%, respectively. In case of a severe looking transport accident these probabilities were 1%, 16%, and 83%, respectively.

**Fig 2:**
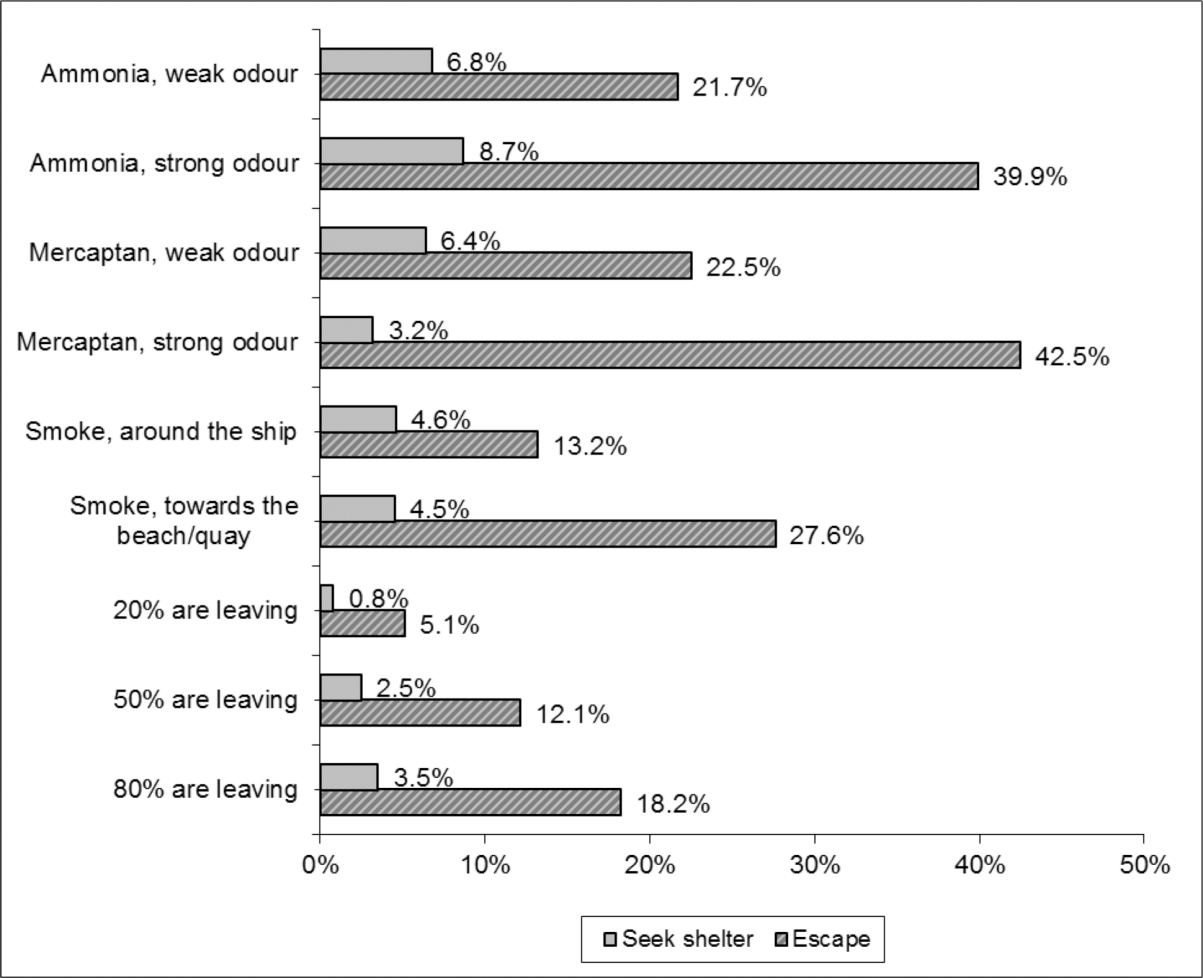
Effects of changing one of the attribute levels on the average probability of citizens’ protective behaviour to transport accidents involving hazardous materials on a populated waterway, as predicted by a panel error component.

Fig 2 shows–keeping all other attribute levels equal—that a perception of a strong ammonia odour had a substantial influence on the predicted probability that a respondent will seek shelter or escape more (9% and 40% more, respectively) compared to ‘no odour perception’. That is, the proportion of people that preferred staying instead of leaving decreased from 62% to 13% (i.e., 62% minus 9% more seeking shelter minus 40% more escaping). The proportion of people that preferred staying instead of leaving decreased from 62% to 16% in case of a strong mercaptan perception (i.e., 62% minus 3% more seeking shelter minus 43% more escaping). If smoke/vapour came close to individuals or half of the people in the environment were leaving, individuals that preferred staying decreased from 62% to 30% (i.e., 62% minus 4% more seeking shelter minus 28% more escaping) and 47% (i.e., 62% minus 3% more seeking shelter minus 12% more escaping), respectively.

## Discussion

Our study showed that the predicted probability that a subject would stay ranged from 1% in case of a severe looking accident till 62% in case of a mild looking accident involving hazardous materials at a river in a densely populated area. The transport accident characteristics ‘odour perception’, ‘smoke/vapour perception’, and ‘the proportion of people in the environment that are leaving at their own discretion’, all proved to influence protective behaviour. A perception of a strong ammonia or mercaptan odour had the strongest influence on protective behaviour; the proportion of subjects that preferred staying instead of leaving (i.e. seeking shelter or escaping) decreased from 62% to 13% and 16%, respectively. If smoke/vapour came close to individuals or if fifty percent of the people in the environment were leaving at their own discretion, the proportion of subjects that preferred staying decreased from 62% to 30% and 47%, respectively. In general, ‘escaping’ was much more preferred than ‘seeking shelter’, although stated preference heterogeneity among subjects for these protective behaviour options was substantial. Males were less willing to seek shelter than females, whereas elderly people were more willing to escape than younger people.

The proportions of age groups in our study differed, as expected, from the Dutch general population (i.e. there were larger proportions of elderly people in the study sample compared to the Dutch general population). The explanation is that the state Zeeland does not contain any big cities (all cities are below 40,000 inhabitants), which attract relatively fewer younger adults. The proportions of age groups in our sample may therefore hamper the generalizability of our results. In contrary, the proportions of different educational levels, marital status and gender in our study sample were all quite similar to the Dutch general population, which suggests the generalizability of our DCE results.

There are no previous DCEs investigating how different characteristics of hazardous material transport accidents at a river in a populated area influenced protective behaviour of the general public. However, Winslott [1] conducted a DCE to study people’s preferences in order to estimate the costs and benefits of different configurations of the transport of hazardous materials by rail, and showed that a reduction in the degree of hazardousness increases utility. This result is in line with our finding, which showed that a perception of weak ammonia/ mercaptan odour or smoke/vapour that does not come close to individuals (i.e. a low degree of hazardousness) increased utility (i.e. ‘staying’ was much more preferred in case of a low degree than in case of a high degree of hazardousness). The positive direction of the attribute coefficient ‘the proportion of people in the environment that are leaving at their own discretion’ showed that the more people in the environment were leaving at their own discretion, the more willing the respondent was to leave the transport accident area as well. This result was consistent with our hypothesis and showed, therefore, theoretical validity.

DCEs are increasingly being used in public health to explore trade-offs the general population make between different alternatives to reduce health risk [25,26]. With an acceptable fraction of potential respondents agreed to participate in the experiment and theoretical valid results, this DCE demonstrated its feasibility to elicit the (determinants of) protective behaviour as well as the general population’s willingness to participate in a relatively complex study to weigh up different aspects of a hazardous material transport accident.

Our DCE study provided insights into subjects’ expected responses to a hazardous materials release originating from a ship. An important question is whether subjects’ stated protective behaviour is consistent with actual protective behaviour? Only a handful of studies have investigated the external validity of DCE outcomes [42–51]. Most of these studies showed that subjects behave in reality as they stated they will do in the DCE questionnaire. To our best knowledge, there are no DCEs investigating to what extent actual protective behaviour during transport accidents involving hazardous materials is in agreement with stated protective behaviour of subjects. Nevertheless this uncertainty, Kang et al. [10] demonstrated a significant degree of correspondence between behavioural expectations and much later behaviour under quite stressful conditions.

The present study had several limitations. First, we selected the most relevant attributes in our DCE using a focus group study and literature, but this careful procedure does not guarantee that attributes that we did not include are irrelevant to citizens’ protective behaviour to transport accidents involving hazardous materials. Second, the inclusion of quantitative information in our DCE might have caused interpretation problems with regard to the choice task. The environmental cues (which had stronger effects) were expressed as words and pictograms, while the social cue (which had a weaker effect) was expressed as numbers and pictograms. Numeracy problems might have affected the results, although 93% of the respondents reported that they did not find the DCE questions difficult. Third, although the response rate of 44% was higher than expected and similar to other DCE studies [52–55], this response rate is still not optimal. We cannot exclude self-selection bias, although our respondents did not differ from non-respondents in age and gender. Additionally, the proportions of different educational levels in our study sample were quite similar with the general population. Finally, the degree of correspondence between mercaptan or ammonia concentrations (e.g. in parts per million) and our verbal labels (strong, weak, or none) of olfactory perceptions is unknown. As people vary in their olfactory sensitivity for a given ambient gas concentration, this issue should be taken into account when actual protective behaviour will be compared to expected protective behaviour.

In conclusion, various characteristics of transport accident involving hazardous materials influence subjects’ protective behaviour. The preference heterogeneity shows that information needs to be targeted differently depending on gender and age to prepare citizens properly.

## Supporting Information

S1 File Translation Questionnaire DCE Westerscheldde.docx. (DOCX)

S2 File FAQ DCE Westerschelde 18-5-2015.docx. (DOCX)

S1 Table The influence of characteristics of hazardous material transport accidents on citizens’ protective behaviour based on a panel error component model including demographic variables (n = 881). (DOCX)
