# Supplementary material for: Protective Behaviour of Citizens to Transport Accidents Involving Hazardous Materials: A Discrete Choice Experiment Applied to Populated Areas nearby Waterways
Source: PLoS One. 2015 Nov 16;10(11):e0142507. doi: 10.1371/journal.pone.0142507 (PMC4646354; doi:10.1371/journal.pone.0142507)
Supplement: S1 File — (DOCX) [file pone.0142507.s001.docx]

| General questions | | | | | | | | | |  |
| --- | --- | --- | --- | --- | --- | --- | --- | --- | --- | --- |
|  | | | | | | | | | |  |
| **A1** | **What is your gender?** | | | | Male  Female | | | | |  |
|  |  | | | |  | | | | |  |
| **A2** | **What is your year of birth?** | | | 19 | | | | | |  |
|  |  | | |  | | | | | |  |
| **A3** | **What are the four digits of your postal code?** | | |  | | | | | |  |
|  |  | | | |  | | | | |  |
|  |  | | | |  | | | | |  |
| **A4** | **What is your marital status?** | | | | Married / registered partnership  Living together  Single, never been married  Divorced, separated living  Widow, widower | | | | |  |
|  |  | | | |  | | | | |  |
| **A5** | **Of how many people does the household consist to which you belong, including yourself?** | | | |  | | *person or persons*  *(including myself)* | | |  |
|  |  | | | | | | | | |  |
| **A6** | **What is your household composition?**Cross one answer on each line. | | | | | | | | |  |
|  |  | | | | | | | **Yes** | **No** |  |
|  | a. | With a partner | | | | | |  |  |  |
|  | b. | With child/children up to 3 years | | | | | |  |  |  |
|  | c. | With child/children 4 to 11 years | | | | | |  |  |  |
|  | d. | With child/children 12 to 17 years | | | | | |  |  |  |
|  | e. | With child/children 18 years or more | | | | | |  |  |  |
|  | f. | With my parent/parents | | | | | |  |  |  |
|  | g. | With other adult/adults | | | | | |  |  |  |
|  | h. | Living Apart Together | | | | | |  |  |  |
|  |  | | | | | | | | |  |
|  |  | | | | | | | | |  |
| **A7** | **What is the highest educational level you have attained?** | | | | | | | | |  |
|  | No education (primary education not completed) | | | | | | | | |  |
|  | Primary school | | | | | | | | |  |
|  | Lower or pre-vocational education (such as LTS, LEAO, LHNO, VMBO) | | | | | | | | |  |
|  | General secondary education (such as MAVO, (M) ULO, MBO short, VMBO-t) | | | | | | | | |  |
|  | Secondary vocational education (such as MBO long, MTS, MEAO, BOL, BBL, INAS) | | | | | | | | |  |
|  | Higher general and pre-university education (such as HAVO, VWO, Atheneum, Gymnasium, HBS, MMS) | | | | | | | | |  |
|  | Higher vocational education (such as HBO, HTS, HEAO, bachelor academic education) | | | | | | | | |  |
|  | University | | | | | | | | |  |
|  | Other, namely | |  | | | | | | |  |
|  |  | | | | | | | | |  |
|  |  | | | | |  | | | | |

| **Questions about behavior in case of an incident** |
| --- |

Important: Please read the information and instructions carefully before answering the questions.

**Information**

**What is meant by transport of hazardous material on the river Westerschelde?**

Hazardous materials are transported on the river Westerschelde. Both toxic (poisonous) and flammable substances are transported. These ships navigate right along the shoreline of the city Vlissingen. When a collision occurs, hazardous material can be released through a hole in the ship. The time between the disaster and its consequences for the people on the boulevard and the beach of the city Vlissingen is very short (minutes).

**Instruction**

**How would you act when you see a disaster with hazardous material on the river Westerschelde?**

In this study it is assumed that people react in different ways when a disaster with hazardous material on the river Westerschelde should occur.

Seek shelter means that you go to a nearby building, such as a shop or hotel on the boulevard (windows and doors closed as much as possible), to protect yourself against hazardous gases.

Escape means that you bring yourself to safety by quickly walking/running away from the disaster (distance more than 300 meters).

The following twelve situations differ from each other. Read the questions as accurately as possible and mark what comes to your mind first. Choose what you probably are going to do. This does not necessarily correspond with whatever is wisely. There are no right or wrong answers.


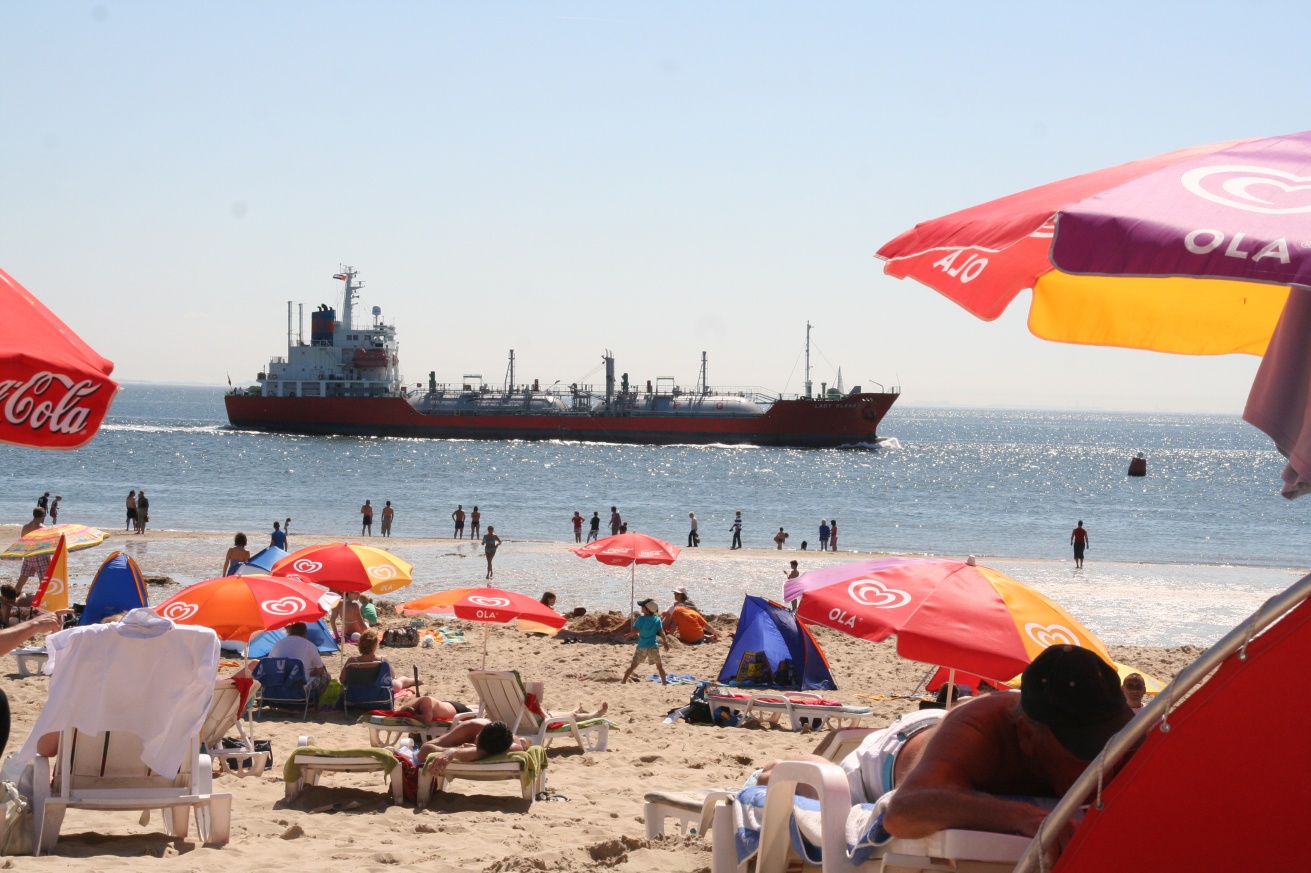


Figure 1: Shipping along the city Vlissingen Photo: Veiligheidsregio Zeeland

| **F1** | On a summer day you are on the boulevard of the city Vlissingen (near Hotel Arion) and see a collision between two ships on the river Westerschelde. The distance between the ships and the boulevard is about 300 meters. There are about a hundred people present on the beach and the boulevard. | | | | | | |
| --- | --- | --- | --- | --- | --- | --- | --- |
| a. |  | **Situation 1** | | | | | |
|  | Odor perception* | Gas smell,  strong odor | | ☹ | | | |
|  | Smoke / vapour observable | No | |  | | | |
|  | Percentage of people that go away (i.e., seek shelter or escape) | 20% | | 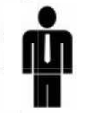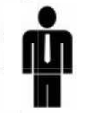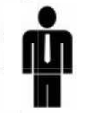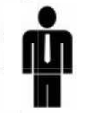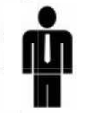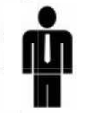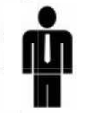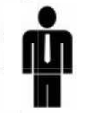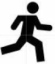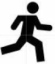 | | | |
|  |  | | | | | | |
|  | **What will you do?**  *Please cross one box.* | | Stay | | Seek shelter | Escape |  |

***Legend:** ☹ = strong odor , 😐 = weak odor, ☺ = no odor


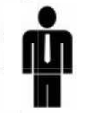
= Stay,
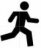
= Escape or seek shelter

| b. |  | **Situation 2** | | | | |
| --- | --- | --- | --- | --- | --- | --- |
|  | Odor perception | Gas smell,  strong odor | | ☹ | | |
|  | Smoke / vapour observable | Yes, around the ship | |  | | |
|  | Percentage of people that go away (i.e., seek shelter or escape) | 20% | | 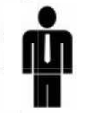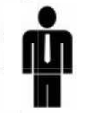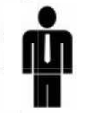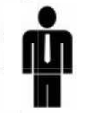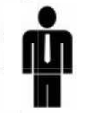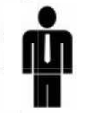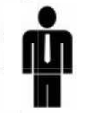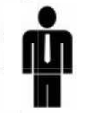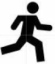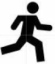 | | |
|  |  | | | | | |
|  | **What will you do?**  *Please cross one box.* | | Stay | | Seek shelter | Escape |

| c. |  | **Situation 3** | | | | |
| --- | --- | --- | --- | --- | --- | --- |
|  | Odor perception | No odor | | ☺ | | |
|  | Smoke / vapour observable | Yes, around the ship | |  | | |
|  | Percentage of people that go away (i.e., seek shelter or escape) | 50% | | 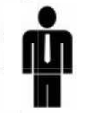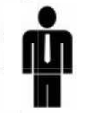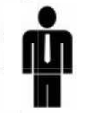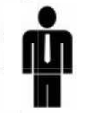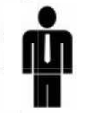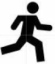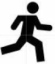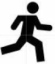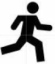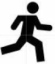 | | |
|  |  | | | | | |
|  | **What will you do?**  *Please cross one box.* | | Stay | | Seek shelter | Escape |

| d. |  | **Situation 4** | | | | |
| --- | --- | --- | --- | --- | --- | --- |
|  | Odor perception | Gas smell,  weak odor | | 😐 | | |
|  | Smoke / vapour observable | No | |  | | |
|  | Percentage of people that go away (i.e., seek shelter or escape) | 80% | | 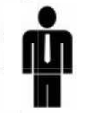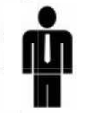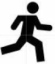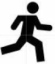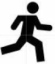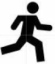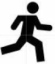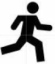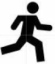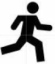 | | |
|  |  | | | | | |
|  | **What will you do?**  *Please cross one box.* | | Stay | | Seek shelter | Escape |

| e. |  | **Situation 5** | | | | |
| --- | --- | --- | --- | --- | --- | --- |
|  | Odor perception | No odor | | ☺ | | |
|  | Smoke / vapour observable | Yes, towards the beach | |  | | |
|  | Percentage of people that go away (i.e., seek shelter or escape) | 50% | | 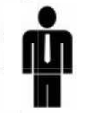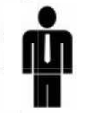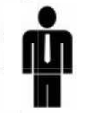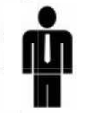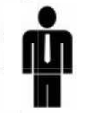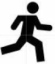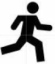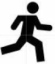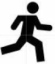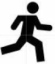 | | |
|  |  | | | | | |
|  | **What will you do?**  *Please cross one box.* | | Stay | | Seek shelter | Escape |

| f. |  | **Situation 6** | | | | |
| --- | --- | --- | --- | --- | --- | --- |
|  | Odor perception | Ammonia smell, weak odor | | 😐 | | |
|  | Smoke / vapour observable | Yes, towards the beach | |  | | |
|  | Percentage of people that go away (i.e., seek shelter or escape) | 0% | | 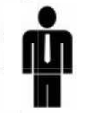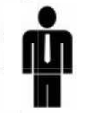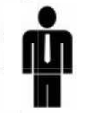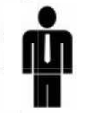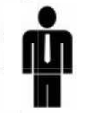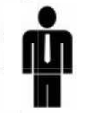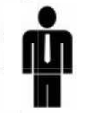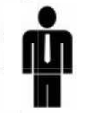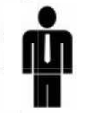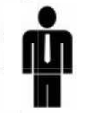 | | |
|  |  | | | | | |
|  | **What will you do?**  *Please cross one box.* | | Stay | | Seek shelter | Escape |

| g. |  | **Situation 7** | | | | |
| --- | --- | --- | --- | --- | --- | --- |
|  | Odor perception | Ammonia smell, strong odor | | ☹ | | |
|  | Smoke / vapour observable | Yes, around the ship | |  | | |
|  | Percentage of people that go away (i.e., seek shelter or escape) | 0% | | 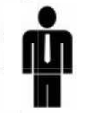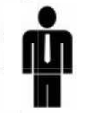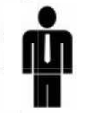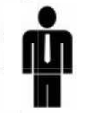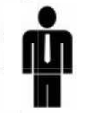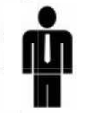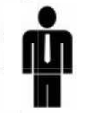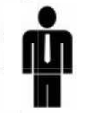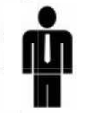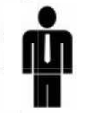 | | |
|  |  | | | | | |
|  | **What will you do?**  *Please cross one box.* | | Stay | | Seek shelter | Escape |

| h. |  | **Situation 8** | | | | |
| --- | --- | --- | --- | --- | --- | --- |
|  | Odor perception | Gas smell,  weak odor | | 😐 | | |
|  | Smoke / vapour observable | Yes, around the ship | |  | | |
|  | Percentage of people that go away (i.e., seek shelter or escape) | 20% | | 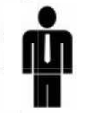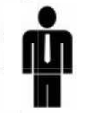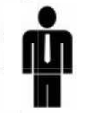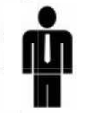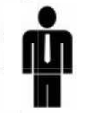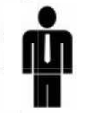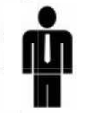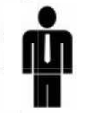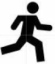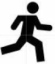 | | |
|  |  | | | | | |
|  | **What will you do?**  *Please cross one box.* | | Stay | | Seek shelter | Escape |

| i. |  | **Situation 9** | | | | |
| --- | --- | --- | --- | --- | --- | --- |
|  | Odor perception | Gas smell,  weak odor | | 😐 | | |
|  | Smoke / vapour observable | Yes, towards the beach | |  | | |
|  | Percentage of people that go away (i.e., seek shelter or escape) | 0% | | 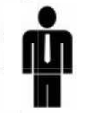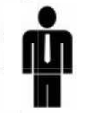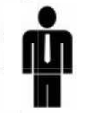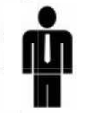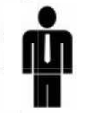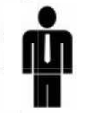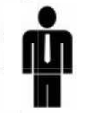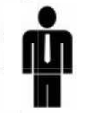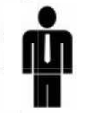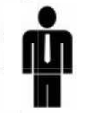 | | |
|  |  | | | | | |
|  | **What will you do?**  *Please cross one box.* | | Stay | | Seek shelter | Escape |

| j. |  | **Situation 10** | | | | |
| --- | --- | --- | --- | --- | --- | --- |
|  | Odor perception | Ammonia smell, weak odor | | 😐 | | |
|  | Smoke / vapour observable | Nee | |  | | |
|  | Percentage of people that go away (i.e., seek shelter or escape) | 80% | | 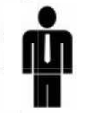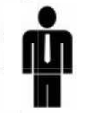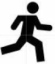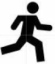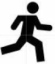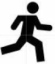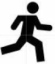 | | |
|  |  | | | | | |
|  | **What will you do?**  *Please cross one box.* | | Stay | | Seek shelter | Escape |

| k. |  | **Situation 11** | | | | |
| --- | --- | --- | --- | --- | --- | --- |
|  | Odor perception | Gas smell,  weak odor | | 😐 | | |
|  | Smoke / vapour observable | Yes, around the ship | |  | | |
|  | Percentage of people that go away (i.e., seek shelter or escape) | 80% | |  | | |
|  |  | | | | | |
|  | **What will you do?**  *Please cross one box.* | | Stay | | Seek shelter | Escape |

| l. |  | **Situation 12** | | | | |
| --- | --- | --- | --- | --- | --- | --- |
|  | Odor perception | Ammonia smell, weak odor | | 😐 | | |
|  | Smoke / vapour observable | Yes, around the ship | |  | | |
|  | Percentage of people that go away (i.e., seek shelter or escape) | 50% | |  | | |
|  |  | | | | | |
|  | **What will you do?**  *Please cross one box.* | | Stay | | Seek shelter | Escape |
