# Supplementary material for: Protective Behaviour of Citizens to Transport Accidents Involving Hazardous Materials: A Discrete Choice Experiment Applied to Populated Areas nearby Waterways
Source: PLoS One. 2015 Nov 16;10(11):e0142507. doi: 10.1371/journal.pone.0142507 (PMC4646354; doi:10.1371/journal.pone.0142507)
Supplement: S2 File — (DOCX) [file pone.0142507.s002.docx]

**Project ‘Kijk uit op de Westerschelde’**

| **Frequently Asked Questions** | |
| --- | --- |
| **1. General** | |
| **Questions** | **Answer** |
| What is the purpose of the study? | The study focuses on two questions. Firstly, we will examine what people will do if an accident occurs on the Westerschelde with hazardous substances. Secondly, the study is also about whether residents living near the Westerschelde can be more prepared for an accident involving hazardous substances, and if so, what is a good preparation? |
| Who is conducting the survey? | The survey is conducted by the Public Health Service (GGD) Zeeland. The GGD Zeeland has received subsidy for this project from ZonMw (www.zonmw.nl). In order to implement the results of research there is cooperation with municipalities.  The Erasmus University Rotterdam and TNO are involved in this project. The Erasmus University Rotterdam will perform complex statistical analyzes for the GGD. |
| What happens to the results of the study? | The collected data will be processed by researchers from the Public Health Service and the Erasmus University Rotterdam. With these results, the GGD will advise local authorities how they can better prepare citizens for a possible disaster involving hazardous material. The results will be ready mid-2013 and will be available to be consulted by everyone (see www.ggdgezondheidsatlas.nl). Data are presented only in the results of the examination of the total group of people who completed a questionnaire and of subgroups, such as men and women. The results are not reducible to one person. |
|  |  |
| **2. Privacy** | |
| How did you receive my name and adress? | All the inhabitants of a municipality are included in the population database of the municipality. Not everyone can get your name and address from the municipality. The Public Health Service is authorized by the national government to randomly select names and addresses. From the population of the three municipalities in Zeeland 2,000 names and addresses were randomly selected. You are one of the persons selected in this way who have been asked to participate in the study. |
| What does "confidential" mean?  Does other people know what I have entered? | We protect the privacy according to the rules for the protection of personal data (Personal Data Protection Act). The survey data are not linked to your name and address. The answers that you fill in the questionnaire can therefore not be traced back to you in any way. The study is not about individuals. The results of the study are presented by data of groups of people: the overall group of people who completed a questionnaire and of subgroups, such as men and women. Nobody knows what you have filled in. |
| Should I give consent to participate in research? Can I withdraw? | With the return of the questionnaire, you give consent to the processing of the data. It is (technically) not possible to withdraw afterwards, because the answers are separated from name and address. As a result, it is not possible to determine from whom the answers are derived. All registered research data are stored according to legal guidelines. |
|  | |
