# Supplementary material for: Protective Behaviour of Citizens to Transport Accidents Involving Hazardous Materials: A Discrete Choice Experiment Applied to Populated Areas nearby Waterways
Source: PLoS One. 2015 Nov 16;10(11):e0142507. doi: 10.1371/journal.pone.0142507 (PMC4646354; doi:10.1371/journal.pone.0142507)
Supplement: S1 Table — (DOCX) [file pone.0142507.s003.docx]

S1 Table

*The influence of characteristics of hazardous material transport accidents on citizens’* *protective behaviour based on a panel error component model including demographic variables (n=881).*
